# Mathematical Models and Experiments on the Acoustic Properties of Granular Packing Structures (Measurement of Tortuosity in Hexagonal Close-Packed and Face-Centered Cubic Lattices)

**DOI:** 10.3390/ma15207393

**Published:** 2022-10-21

**Authors:** Shuichi Sakamoto, Kyosuke Suzuki, Kentaro Toda, Shotaro Seino

**Affiliations:** 1 Department of Engineering, Niigata University, Ikarashi 2-no-cho 8050, Nishi-ku, Niigata 950-2181, Japan; 2 Graduate School of Science and Technology, Niigata University, Ikarashi 2-no-cho 8050, Nishi-ku, Niigata 950-2181, Japan

**Keywords:** sound absorption coefficient, hexagonal close-packed lattice, face-centered cubic lattice, tortuosity

## Abstract

In this study, the sound absorption characteristics of hexagonal close-packed and face-centered cubic lattices were estimated by theoretical analysis. Propagation constants and characteristic impedances were obtained by dividing each structure into elements perpendicular to the incident direction of sound waves and by approximating each element to a clearance between two parallel planes. Consequently, the propagation constant and the characteristic impedance were treated as a one-dimensional transfer matrix in the propagation of sound waves, and the normal incident sound absorption coefficient was calculated by the transfer matrix method. The theoretical value of the sound absorption coefficient was derived by using the effective density applied to the measured tortuosity. As a result, the theoretical value was becoming closer to the measured value. Therefore, the measured tortuosity is reasonable.

## 1. Introduction

The structures filled with granular materials are used to reduce noise, such as in low-noise pavements [1] and ballast tracks [2], owing to their acoustic characteristics. These continuous clearance structures exhibit acoustic properties similar to those of porous materials, and their acoustic properties change depending on the layer thickness, particle size, and packing structure. Therefore, the prediction of the acoustic properties of the structures filled with granular materials based on the particle size, packing structure, and physical properties of gases is useful for their engineering applications.

Various studies have been conducted on the sound absorption characteristics of granular packing structures, including experimental studies on the acoustic properties of loosely packed granular materials [3], the prediction of the acoustic properties of face-centered cubic structures [4], the numerical analysis of multiple regularly packed structures using commercial software [5], and the study of the sound absorption characteristics of random close-packed materials [6]. In addition, the acoustic properties of granular materials [7], the sound absorption coefficient in a powder bed [8,9], and sound absorption due to gaps in the packing structure of simple cubic and hexagonal lattices [10] have been studied.

In this study, the sound absorption characteristics of hexagonal close-packed and face-centered cubic lattices were estimated by theoretical analysis. Propagation constants and characteristic impedances were obtained by dividing each structure into elements perpendicular to the incident direction of sound waves and approximating each element to a clearance between two parallel planes. Consequently, the propagation constant and the characteristic impedance were treated as a one-dimensional transfer matrix in the propagation of sound waves, and the normal incident sound absorption coefficient was calculated by the transfer matrix method. This method measures the sound absorption coefficient using a one-dimensional (1D) wave equation from simplified geometrical information of the granular material. The program and the number of calculations are so simple that they cannot be compared to the finite element method (FEM), for example, and the calculation can be completed in a fraction of a second on a standard PC.

We also measured the tortuosity, which is used to represent the complexity of the path through which sound waves propagate in the structure. The theoretical value of the sound absorption coefficient was derived by using the effective density applied to the measured tortuosity which is one of the Biot parameters. The drawback of this method may be that the theoretical value includes a measurement of tortuosity, but instead, a simplified method for measuring tortuosity is proposed.

In the measurement of the sound absorption coefficient, the normal incident sound absorption coefficient of each sample was measured using a two-microphone impedance tube. The comparison between experimental and theoretical values was reported.

## 2. Samples and Measuring Device Used to Measure the Sound Absorption Coefficient

### 2.1. Transmission Loss Measurement

**Figure 1:**
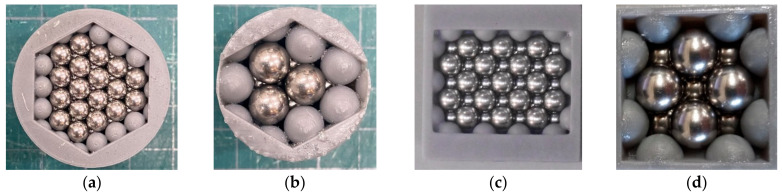
Test samples: (**a**) Hexagonal close-packed lattice (*d* = 4 mm); (**b**) Hexagonal close-packed lattice (*d* = 8 mm); (**c**) Face-centered cubic lattice (*d* = 4 mm); (**d**) Face-centered cubic lattice (*d* = 8 mm).

In this study, two types of packing structures, hexagonal close-packed and face-centered cubic lattices, were investigated. The samples used for the measurement of sound absorption are shown in Figure 1a–d, and the sample specifications are shown in Table 1. Stainless steel spheres with diameters of *d* = 4 mm and 8 mm were used as particles.

In the preparation of the measurement sample, the granular material was packed regularly with the sample holder. The sample holder was regularly arranged with a hemispherical convex part on the wall, and a regular packing structure was formed by placing a granular body in a predetermined position. The sample holder was fabricated in photocurable resin using a photocurable 3D printer Form 2 manufactured by Formlabs Inc. (Somerville, MA, USA).

### 2.2. Measurement Equipment for Sound Absorption Coefficient

**Figure 2:**
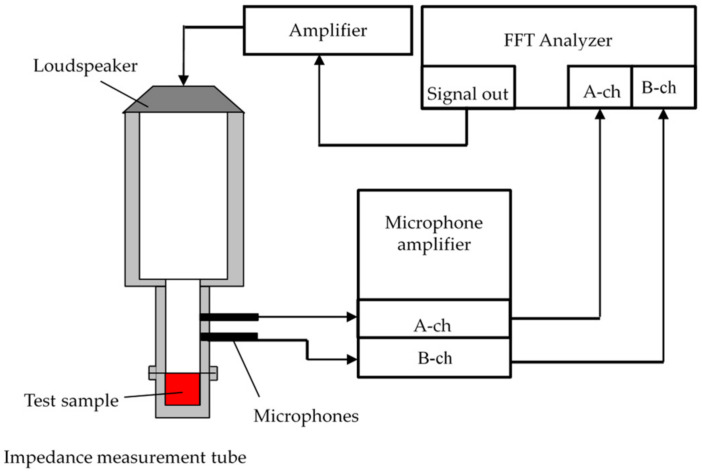
Configuration diagram of a two-microphone impedance tube for the absorption coefficient measurement.

A 4206-type two-microphone impedance tube made by Brüel and Kjær was used to measure the sound absorption coefficient. The configuration of the device is shown in Figure 2. The sample was enclosed in an impedance tube, and a sine wave signal was output by an internal signal generator in the DS-3000 fast Fourier transform (FFT) analyzer manufactured by Ono Sokki, and the transfer function between the sound pressure signals of two microphones attached to the impedance tube was measured by an FFT analyzer. Using the measured transfer function, the normal incident sound absorption coefficient was calculated in accordance with ISO 10534-2. The critical frequency of the plane wave depended on the inner diameter of the acoustic tube. In this study, a small tube with an inner diameter of 29 mm was used because the sound absorption coefficient in the low-frequency range was not high. Therefore, the measurement was performed in the range of 500–6400 Hz.

## 3. Measurement Method and Results of Tortuosity

### 3.1. Overview of Tortuosity

Tortuosity, which is a Biot parameter, is the ratio of the average length of voids in the poroelastic material to the thickness of the sound-absorbing material. When a sound wave passes through a sound-absorbing material with a complicated internal clearance structure, the tortuosity represents the complexity of the path of the sound wave. In this study, the tortuosity for each packed structure was measured using an ultrasonic sensor. In general, the tortuosity *α*_∞_ is expressed as Equation (1) using the sound velocity *c*_0_ in the air and the apparent sound velocity *c* in the packed structure [11]:

(1) $$\alpha_{\infty }={\left(\frac{c_{0}}{c}\right)}^{2}$$

### 3.2. Tortuosity Measurement

The tortuosity was calculated by Equation (1) from the square of the ratio of two sound velocities: the sound velocity in the air without a sample and the sound velocity transmitted through the sample. Ultrasonic waves were output from an ultrasonic sensor on the transmitter side using a sinusoidal signal generated by a signal generator. Ultrasonic waves propagated in the sample were measured using an ultrasonic sensor on the receiving side, and the waveform was observed using an oscilloscope. The propagation time of ultrasonic waves from the transmitter to the receiver was calculated from the comparison between the original waveform obtained by the signal generator and that of ultrasonic waves propagated in the sample, and the apparent sound velocity *c* in the sample in Equation (1) was calculated.

**Figure 3:**
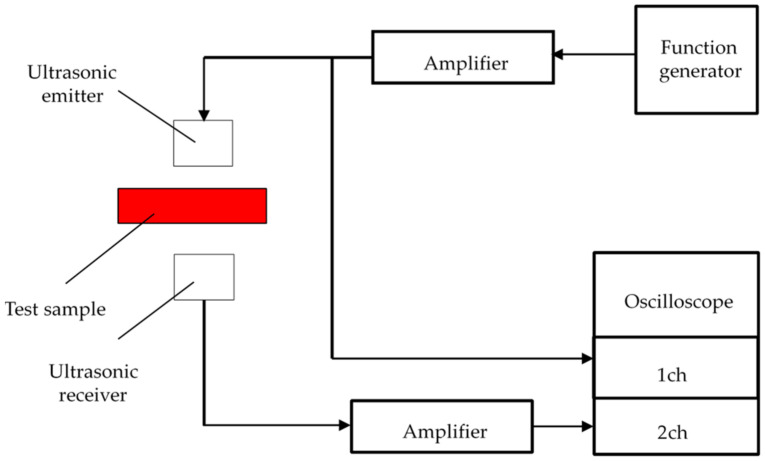
Configuration diagram of the tortuosity measurement.

Figure 3 shows the configuration of the tortuosity measurement device. Ultrasonic sensors with center frequencies of 32.7 kHz, 40 kHz, 58 kHz, 110 kHz, 150 kHz, 200 kHz, and 300 kHz were used. First, the tortuosity *α*_∞_ at each frequency was measured for each packed structure. Then, the reciprocal of the square root of the frequency used for the measurement was determined as the value of the horizontal axis, and the tortuosity *α*_∞_ at each frequency obtained by the measurement was plotted as the value of the vertical axis. The linear approximation of these point clouds leads to a soaring straight line using the least-squares method. When the frequency of the approximate line is set to infinity, the limiting value of the tortuosity, i.e., the *y*-intercept of the graph, becomes the tortuosity *α*_∞_ of the packing structure.

At higher frequencies, ultrasonic sensors have a lower signal-to-noise ratio (S/N ratio or SNR) due to a lower conversion efficiency and a higher attenuation of sound waves in the air. To improve the S/N ratio and measurement accuracy, 150 measurements were added synchronously. The signal was measured in the amplitude direction with a resolution of 16 bits.

**Figure 4:**
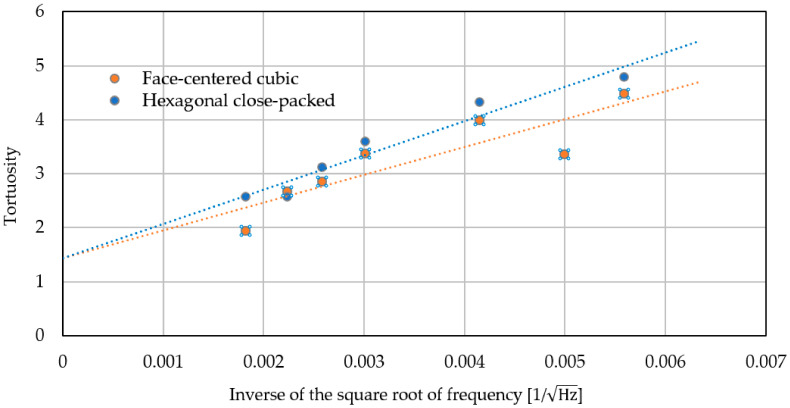
Measurement results of the tortuosity.

Table 2 and Figure 4 show the results of tortuosity measurements. As mentioned above, the *y*-intercept of the approximate straight line is the tortuosity *α*_∞_ of each packing structure. At 40 kHz, the deviation was excluded for the hexagonal structure. The tortuosity of each packed structure was 1.44 for the hexagonal close-packed structure and 1.43 for the face-centered cubic lattice.

Generally, measuring tortuosity requires expensive equipment, and outsourcing the measurement is not cost-effective. In addition, experimental methods with very high ultrasonic frequencies make it difficult to measure tortuosity on rigid granular objects such as those used in this study (where the rigid surfaces have a significant size relative to the wavelength) because ultrasonic waves are reflected.

To avoid the abovementioned problems, this apparatus uses several ultrasonic transducers with low frequencies. Other devices are very common. This simple method of measuring tortuosity may be useful for rigid granular materials such as those used in this study.

## 4. Theoretical Analysis

### 4.1. Analysis Units and Element Division

**Figure 5:**
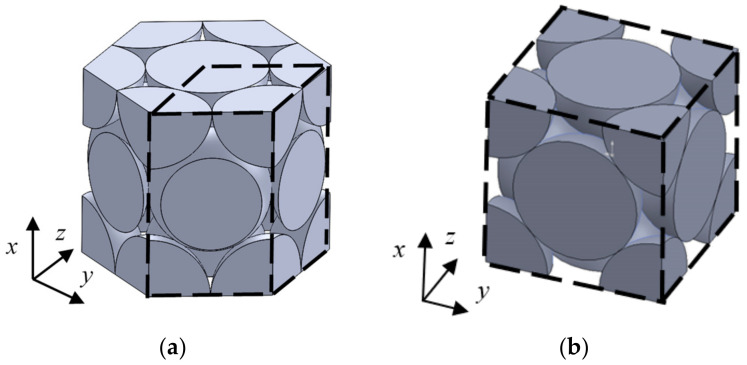
Analysis unit in each structure: (**a**) Hexagonal close-packed lattice; (**b**) Face-centered cubic lattice.

**Figure 6:**
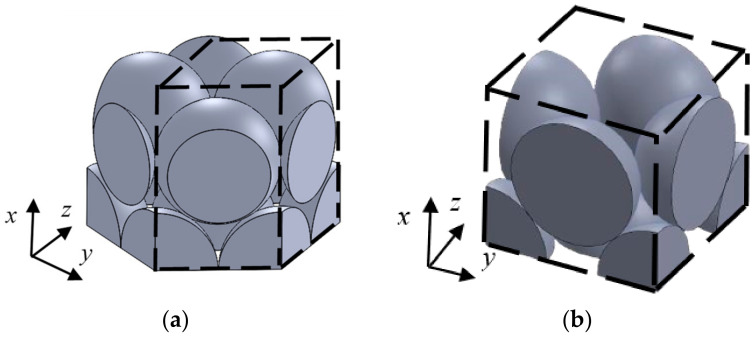
Analysis unit at the top of the sample: (**a**) Hexagonal close-packed lattice; (**b**) Face-centered cubic lattice.

The gap in the packed structure of granular materials was analyzed by the transfer matrix method based on the one-dimensional wave equation. In this section, the outline of the theoretical analysis is explained. Figure 5 shows the analysis units for each packing structure. In these structures, the cross-sectional shape changes periodically in the *x*-, *y*-, and *z*-axis directions. The range, whereby the change in the cross-sectional shape was completed in one cycle, was used as the analysis unit. Namely, the analysis unit was defined as the area surrounded by the broken line in Figure 5. In addition, the analysis unit located at the upper edge of the sample in Figure 5a,b (i.e., the incident surface of the sound wave) in the *x* direction is shaped, as shown in Figure 6a,b, respectively.

**Figure 7:**
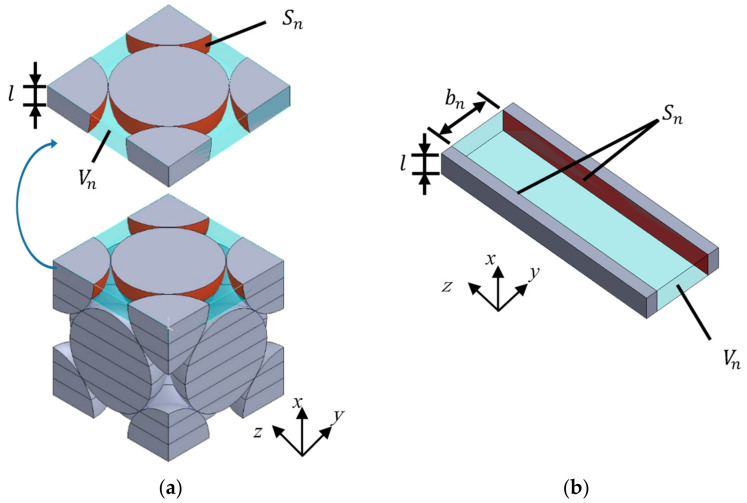
Divided element approximated to the clearance between two planes: (**a**) Divided element (face-centered cubic); (**b**) Approximated clearance between two planes.

In the packing structure of the granular materials, the shape of the cross-section changes continuously in the plane perpendicular to the direction of sound wave propagation. Therefore, the analysis unit was divided into 100 parts in the direction perpendicular to the *x*-axis. Figure 7a shows the analysis unit division method for the face-centered cubic lattice shown in Figure 5b. The analysis units shown in Figure 5a and Figure 6a,b were also divided, as in the case of Figure 7a. The number of divisions of the analysis unit, *n* = 100, is the value at which the theoretical value of the normal incident sound absorption coefficient converges.

For the clearance between particles in the divided element shown in Figure 7a, the real surface area *S_n_* and real volume *V_n_* of the clearance were geometrically calculated and approximated to the thickness of the clearance between two planes *b_n_*, as shown in Figure 7b and Equation (2).

**Figure 8:**
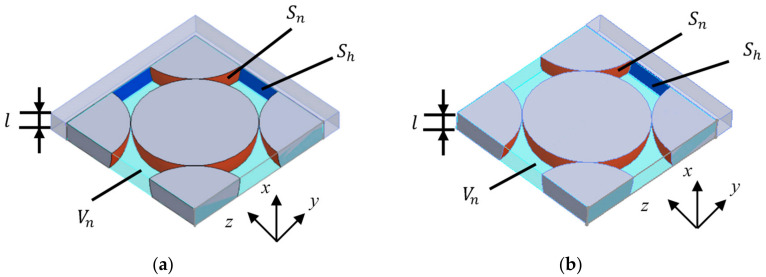
Approximated clearance between two planes at the element in contact with the sample holder: (**a**) Divided element in contact with the sample holder on two sides; (**b**) Divided element in contact with the sample holder on one side.

For the divided elements in contact with the sample holder, as shown in Figure 8a,b, the area *S_h_* of the wall of the sample holder was also taken into consideration, and it was approximated to the clearance between two planes. As shown in Figure 8a,b, when the clearance thickness is set to *b_n_*’, *S_n_*, *S_h_*, and *V_n_* are expressed as Equation (3):

(2) $$b_{n}=\frac{2V_{n}}{S_{n}}$$

(3) $$b_{n}\prime =\frac{2V_{n}}{S_{n}+S_{h}}$$

### 4.2. Derivation of the Surface Area of a Sphere in a Divided Element

In this section, we describe the derivation method of the surface area *S_n_* of spheres in the divided element for the hexagonal close-packed and face-centered cubic lattices used in Equations (2) and (3).

Using the radius *r* of the sphere and the length *Z_unit_* of the dividing element in the *x*-axis direction, the surface area *S_n_* of the sphere at the dividing element can be obtained by Equation (4):

(4) $$S_{n}=2\pi r\times Z_{unit}$$

As shown in Figure 8, the area *S_h_* of the blue portion of the sample holder wall was considered for the divided element in contact with the sample holder.

### 4.3. Derivation of the Volume of the Clearance in a Divided Element

In this section, we describe the derivation method of the volume *V_n_* of clearances in the divided element of the hexagonal close-packed and face-centered cubic lattice structures.

*V_n_*, for the hexagonal close-packed structure, shows the clearance in the range surrounded by the broken line in Figure 5a and is derived geometrically using the dimensions of the radius *r* of the sphere and the length *Z_unit_* of the divided element in the *x*-axis direction, as follows:

(5) $$V_{n}=2\sqrt{3}r^{2}\times Z_{unit}-2\int \pi \left(r^{2}-x^{2}\right)dx$$

*V_n_*, for the face-centered cubic structure, shows the clearance in the range surrounded by the broken line in Figure 5b, which is geometrically derived as follows:

(6) $$V_{n}=8r^{2}\times Z_{unit}-4\int \pi \left(r^{2}-x^{2}\right)dx$$

### 4.4. Propagation Constant and Characteristic Impedance Considering Tortuosity

Propagation constants and characteristic impedances in the clearance between two planes approximated in Section 4.1 were obtained by considering the attenuation of sound waves. Propagation constants and characteristic impedances considering the viscosity of the air in tubes were studied by Tijdeman [12] and Stinson [13] for circular tubes, Stinson [14] for equilateral triangles, and Beltman [15] for rectangular tubes. In addition, Allard [16] considered the effect of tortuosity. In this study, the methods reported by Stinson [14] and Allard [16] were applied.

**Figure 9:**
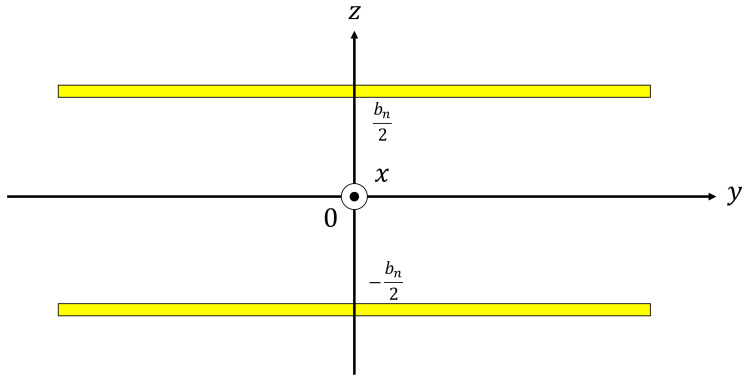
Cartesian coordinate system for the parallel clearance between the two planes.

The Cartesian coordinate system was used, as shown in Figure 9, and the effective density *ρ_s_* and compressibility *C_s_* were obtained from a three-dimensional analysis using Equations (7) and (8) [14], respectively, using the Navier–Stokes equations, the equation of state of gas, the continuity equation, the energy equation, and the dissipation function representing heat transfer, where *ρ*_0_ is the density of the air, *λ_s_* is an intermediary variable, *b_n_* is the clearance thickness between two planes, *ω* is the angular frequency, *η* is the viscosity of the air, *κ* is the specific heat ratio of the air, *P*_0_ is the atmospheric pressure, and *N_pr_* is the Prandtl number.

(7) $$\rho_{s}=\rho_{0}{\left[1-\frac{\tanh\left(\sqrt{j}\lambda_{s}\right)}{\sqrt{j}\lambda_{s}}\right]}^{-1},\lambda_{s}=\frac{b_{n}}{2}\sqrt{\frac{\omega \rho_{0}}{\eta }}$$

(8) $$C_{s}=\left(\frac{1}{\kappa P_{0}}\right)\left\{1+\left(\kappa -1\right)\left[\frac{\tanh\left(\sqrt{jN_{pr}}\lambda_{s}\right)}{\sqrt{jN_{pr}}\lambda_{s}}\right]\right\}$$

By using the effective density *ρ_s_* multiplied by the tortuosity *α*_∞_, the propagation constant and characteristic impedance considering the tortuosity can be obtained [16]. Therefore, the propagation constant *γ* and the characteristic impedance *Z_c_* can be expressed by the following equations [16] in terms of the effective density *ρ_s_* and compressibility *C_s_* when the tortuosity *α*_∞_ is considered:

(9) $$\gamma =j\omega \sqrt{\alpha_{\infty }\rho_{s}C_{s}}$$

(10) $$Z_{c}=\sqrt{\frac{\alpha_{\infty }\rho_{s}}{C_{s}}}$$

### 4.5. Transfer Matrix

**Figure 10:**
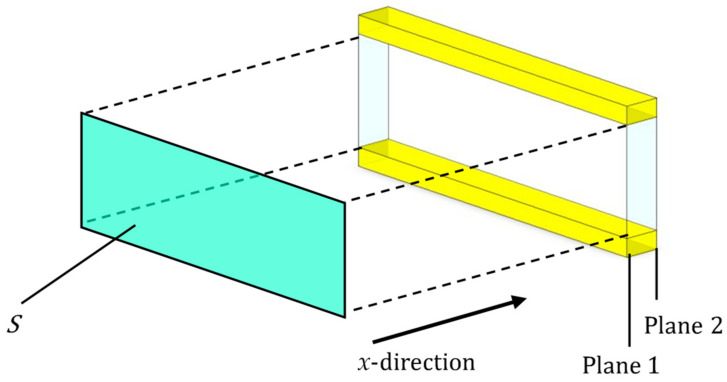
Sound incident area, incident plane, and transmission plane of approximated clearance between two planes in Figure 7b and Figure 9.

The clearance between two planes was analyzed by the transfer matrix method with respect to sound pressure and volumetric velocity based on the one-dimensional wave equation. Figure 10 shows a schematic diagram showing one element in the *x*-direction for the clearance between two planes shown in Figure 9. Using the characteristic impedance, the propagation constant; the cross-sectional area of the clearance, *S*; and the length of the divided element, *l*, obtained in Section 4.1,Section 4.2,Section 4.3 and Section 4.4 the transfer matrix, *T_n_*; and the four-terminal constants of the acoustic tube element, *A* to *D*, can be calculated using Equation (11):

(11) $$T_{n}=\left[\begin{array}{cc} \cosh\left(\gamma l\right) & \frac{Z_{c}}{S}sinh\left(\gamma l\right)\\ \frac{S}{Z_{c}}sinh\left(\gamma l\right) & \cosh\left(\gamma l\right) \end{array}\right]=\left[\begin{array}{cc} A & B\\ C & D \end{array}\right]$$

Plane 1 is the incident surface of sound waves, and Plane 2 is the transmission surface of sound waves. The sound pressure and the particle velocity can be expressed as *p*_1_ and *u*_1_ at Plane 1 and *p*_2_ and *u*_2_ at Plane 2, respectively, and the transfer matrix can be expressed as Equation (12):

(12) $$\left[\begin{array}{c} p_{1}\\ Su_{1} \end{array}\right]=\left[\begin{array}{cc} A & B\\ C & D \end{array}\right]\left[\begin{array}{c} p_{2}\\ Su_{2} \end{array}\right]$$

**Figure 11:**
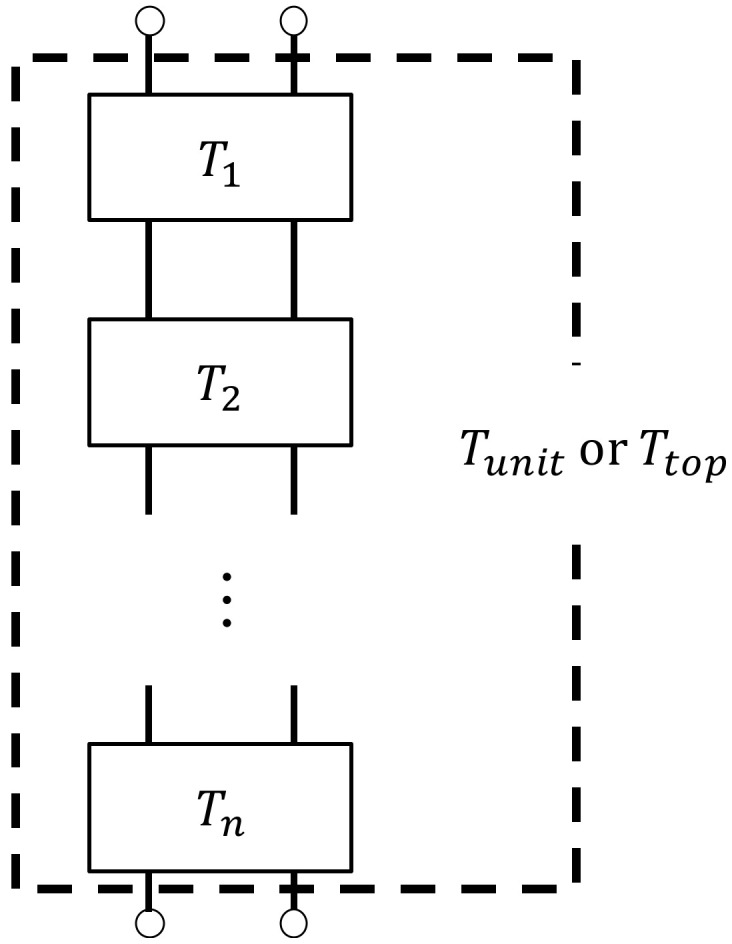
Equivalent circuit in the analysis unit (cascade connecting the transfer matrix of each element).

When Equation (12) was applied to the clearance between the two planes obtained in Section 4.2 and Section 4.3, the transfer matrix was obtained at each divided element. Since each divided element is continuous in the *x*-axis direction, the transfer matrix *T_unit_* and *T_top_* of the analysis unit and the analysis unit at the upper edge of the sample were obtained by cascading the transfer matrix of each divided element based on the equivalent circuit shown in Figure 11.

**Figure 12:**
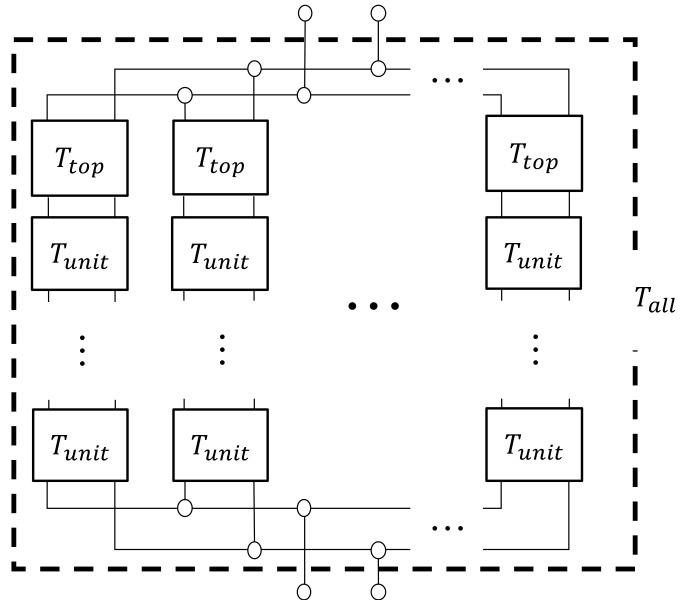
Equivalent circuit of the whole sample (parallel connection of the cascaded *T_unit_*).

Next, as shown in Figure 12, the transfer matrix corresponding to the analysis unit was connected based on the equivalent circuit of the whole sample, and the transfer matrix of the whole sample was derived.

First, the analysis unit that was arranged in the *x*-axis direction, i.e., the incident direction of sound waves, was cascaded. Next, the transfer matrix of the whole sample was obtained by connecting the cascade-connected transfer matrix that aligned on the *y*–*z* plane, the plane perpendicular to the incident direction of the sound wave, in parallel.

### 4.6. Normal Incident Sound Absorption Coefficient

The sound absorption coefficient was calculated from the transfer matrix *T_all_* obtained in Section 4.5. Since the end of the specimen used in this study was a rigid wall, the particle velocity *u*_2_ = 0, Equation (12), can be deformed as shown in Equation (13), yielding Equation (14):

(13) $$\left[\begin{array}{c} p_{1}\\ Su_{1} \end{array}\right]=\left[\begin{array}{cc} A & B\\ C & D \end{array}\right]\left[\begin{array}{c} p_{2}\\ 0 \end{array}\right]$$

(14) $$\left[\begin{array}{c} p_{1}\\ Su_{1} \end{array}\right]=\left[\begin{array}{c} Ap_{2}\\ Cp_{2} \end{array}\right]$$

When the sound pressure and the particle velocity just outside of Plane 1 are *p*_0_ and *u*_0_, the specific acoustic impedance *Z*_0_ from the incident surface of the sample is expressed as follows:

(15) $$Z_{0}=\frac{p_{0}}{u_{0}}$$

Therefore, according to *p*_0_ = *p*_1_, *S*_0_*u_0_* = *Su*_1_, and Equation (15), the specific acoustic impedance *Z*_0_ of the sample is expressed as

(16) $$Z_{0}=\frac{p_{0}}{u_{0}}=\frac{p_{0}}{u_{0}S_{0}}S_{0}=\frac{p_{1}}{u_{1}S}S_{0}=\frac{A}{C}S_{0}$$

where *S/S*_0_ is the aperture ratio of the sample shown in Table 1.

The relation between the specific acoustic impedance *Z*_0_ and the reflectance *R* is expressed by the following equation:

(17) $$R=\frac{Z_{0}-\rho_{0}c_{0}}{Z_{0}+\rho_{0}c_{0}}$$

According to Equation (17), the theoretical value of the normal incident sound absorption coefficient *α* of the sample is expressed as follows:

(18) $$\alpha =1-{\left|R\right|}^{2}$$

## 5. Comparison between Measured and Theoretical Values

**Figure 13:**
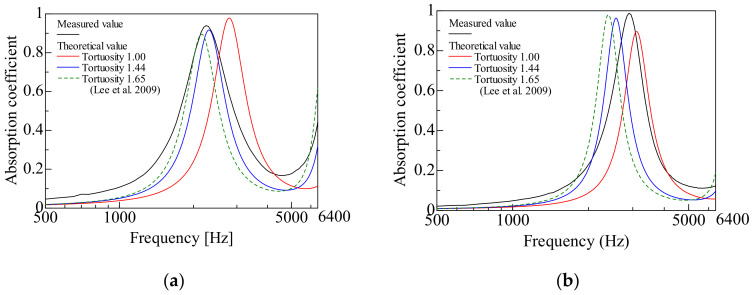

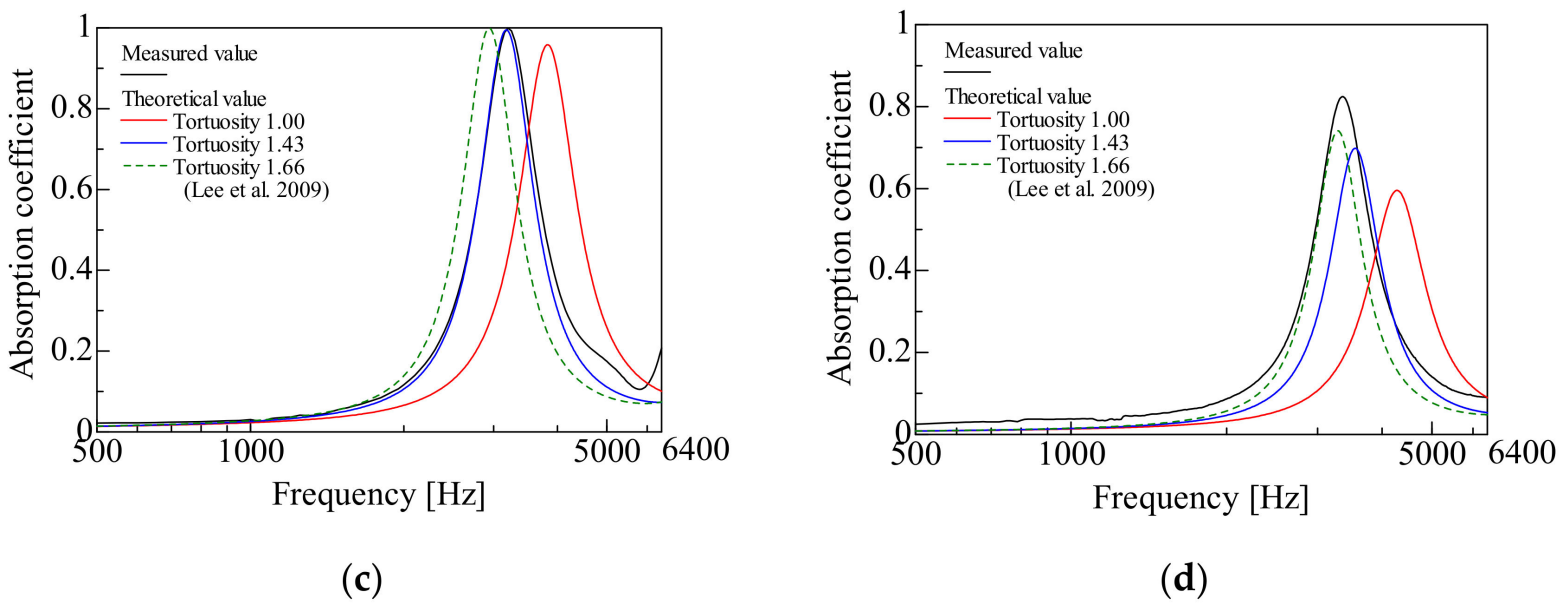
Comparison between the experimental and calculated values (considering tortuosity in Section 3.2 and tortuosity in Reference (Lee et al. 2009) [5]) of the peak frequency and the sound absorption coefficient: (**a**) Hexagonal close-packed lattice (*d* = 4 mm); (**b**) Hexagonal close-packed lattice (*d* = 8 mm); (**c**) Face-centered cubic lattice (*d* = 4 mm); and (**d**) Face-centered cubic lattice (*d* = 8 mm).

In this section, the theoretical value of the normal incident sound absorption coefficient is compared with the measured value for each sample. Three theoretical values are presented for Figure 13a–d: first, for the case where the tortuosity is not considered (unity); for the tortuosity measured in Section 3.2 (hexagonal close-packed lattice: 1.44, face-centered cubic lattice: 1.43); and for the sound absorption coefficient which derived from the tortuosity obtained by the numerical analysis in [5] (HCP: 1.65, FCC: 1.66). Here, the tortuosity measured in Section 3.2 shows smaller values than those obtained from the numerical analysis in [5].

Figure 13a,b show the hexagonal close-packed structure, and Figure 13c,d show the comparison of the face-centered cubic lattice with grain sizes of *d* = 4 mm and *d* = 8 mm, respectively.

First, we compared the measured values with theoretical values without considering the tortuosity. As shown in Figure 13a–d, the peak frequency of the theoretical value, which did not consider the tortuosity, compared with the measured value that appeared on the high-frequency side. Except for Figure 13a, the theoretical sound absorption peak values, without considering the tortuosity, were lower than the measured values.

Next, we focused on the theoretical values that considered the tortuosity. The peak frequency shifted to a lower frequency in Figure 13a–d, and the peak sound absorption value increased in all cases (except for Figure 13a).

As a result, the difference between the theoretical and measured values decreased when the tortuosity was considered. A larger tortuosity than unity means a longer path of sound waves that propagate in the sample, similar to the increase in sample thickness. Therefore, it is considered that the peak frequency of the theoretical value moved to the low-frequency side.

In general, the sound absorption coefficient of porous materials is greatly influenced by thickness [17,18]. In other words, the sound absorption peak occurs at a frequency where the thickness of the porous sound-absorbing material corresponds to one-quarter wavelength of the sound wave. Conversely, if the apparent sound velocity in the material decreases because of boundary layer friction or tortuosity, the peak sound absorption frequency will decrease, as this is equivalent to an increase in the apparent thickness of the material. That is, an increase in tortuosity decreases the peak sound absorption frequency and increases the sound absorption curve in most cases. For the same reason, in Figure 13b–d, it is considered that the theoretical sound absorption peak value increased, owing to the consideration of the tortuosity.

Next, we discuss the tortuosity in each sample. As shown in Section 3, the measured tortuosities in the hexagonal close-packed and face-centered cubic lattices were 1.44 and 1.43, respectively.

By considering one of the two types of tortuosities, the theoretical values of the peak frequency and the sound absorption peak were in good agreement with the measured values for the hexagonal close-packed lattice with a grain size *d* = 4 mm, as shown in Figure 13a,b. Here, the theoretical values, considering the measured tortuosity in Section 3.2, are closer to the experimental values. However, when the grain size of the hexagonal close-packed structure was *d* = 8 mm, the peak frequency shifted to a lower frequency with the consideration of either of the tortuosities, but it was lower than the peak frequency side of the measured value because the division and assembly of the sample holder led to errors in the measured values. For the measured hexagonal close-packed structure with a grain size of *d* = 8 mm, the number of spheres used was small. To avoid interference between the particles during the sample assembly, the holder was divided into three parts perpendicular to the incident direction of the sound wave. Dimensional errors between the divided holders may have caused gaps in the spheres, thereby affecting the measured values. On the other hand, for the grain size *d* = 4 mm, the accuracy of the theoretical value was improved by considering the measured tortuosity in Section 3.2.

In the case of the face-centered cubic lattice with a grain size of *d* = 4 mm, as shown in Figure 13c, the theoretical values were fairly close to the measured values when either tortuosity was considered. When the two types of tortuosities were compared, the theoretical values from the tortuosity measured in Section 3.2 agreed very well with the measured values. When the grain size of the face-centered cubic lattice was *d* = 8 mm, as shown in Figure 13d, by considering either of the tortuosities, the theoretical values of both the peak frequency and the sound absorption peak approached the measured values, but the degree of agreement was smaller than that in Figure 13c. In terms of the agreement between theoretical (applying the tortuosity in Section 3.2) and experimental values, the case with a grain size of 4 mm (Figure 13a,c) is better than the case with a grain size of 8 mm (Figure 13b,d). This is because the method used to estimate the attenuation of sound waves in gaps [14] is more accurate for smaller gaps [19].

## 6. Conclusions

The normal incidence sound absorption coefficients of hexagonal close-packed and face-centered cubic lattices, which are typical packing structures of granular materials, were theoretically calculated. In the derivation of the propagation constant and the characteristic impedance, the measured tortuosity was taken into account. The results were compared with the experimental data. The following conclusions were obtained:

1. In both packing structures, the real area of the granular surface and the real volume of the clearance were obtained geometrically and analyzed theoretically.
2. In both packing structures, the peak frequency tended to appear at a higher frequency than the measured value when the tortuosity was not considered.
3. In the theoretical sound absorption, the peak value was higher when the tortuosity was considered compared to that without the consideration of the tortuosity (the peak frequency moved to a lower frequency). As a result, the theoretical value was becoming closer to the measured value.

## Figures and Tables

**Table 1:** Properties of test samples.

Packing Structure	Diameter [mm]	Length [mm]	Aperture Ratio of Sample Holder	Filling Rate	Measured Tortuosity	Correspondence to Figure
Hexagonal close-packed	4	27	0.57	0.74	1.44	1a
	8	27	0.67	0.74	1.44	1b
Face-centered cubic	4	22	0.58	0.74	1.43	1c
	8	21	0.85	0.74	1.43	1d

**Table 2:** Measurement results of the tortuosity.

Frequency [kHz]	Inverse of the Square Root of Frequency $\left[1/\sqrt{\mathrm{Hz}}\right]$	Distance between Sensors [mm]	Transmission Time [ms]	Tortuosity	
				Hexagonal Close-Packed	Face-Centered Cubic
32.7	0.00559	395	1.229	4.78	4.48
40	0.005	395	-	-	3.35
58	0.004152	395	1.199	4.31	3.98
110	0.003015	345	1.044	3.59	3.36
150	0.002582	204	0.626	3.11	2.84
200	0.002236	204	0.618	2.56	2.67
300	0.001826	204	0.615	2.56	1.93
∞	0	-	-	1.44	1.43

## References

[1] Sandberg U. (1999). Low noise road surfaces—A state-of-the-art review. J. Acoust. Soc. Jpn..

[2] Zhang X., Thompson D., Jeong H., Squicciarini G. (2017). The effects of ballast on the sound radiation from railway track. J. Sound Vib..

[3] Voronina N.N., Horoshenkov K.V. (2003). A new empirical model for the acoustic properties of loose granular media. Appl. Acoust..

[4] Gasser S., Paun F., Bréchet Y. (2005). Absorptive properties of rigid porous media: Application to face centered cubic sphere packing. J. Acoust. Soc. Am..

[5] Lee C.-Y., Leamy M.J., Nadler J.H. (2009). Acoustic absorption calculation in irreducible porous media: A unified computational approach. J. Acoust. Soc. Am..

[6] Dung V.V., Panneton R., Gagné R. (2019). Prediction of effective properties and sound absorption of random close packings of monodisperse spherical particles: Multiscale approach. J. Acoust. Soc. Am..

[7] Sakamoto S., Sakuma Y., Yanagimoto K., Watanabe S. (2012). Basic Study for the Acoustic Characteristics of Granular Material (Normal Incidence Absorption Coefficient for Multilayer with Different Grain Diameters). J. Environ. Eng..

[8] Sakamoto S., Yamaguchi K., Ii K., Takakura R., Nakamura Y., Suzuki R. (2019). Theoretical and experiment analysis on the sound absorption characteristics of a layer of fine lightweight powder. J. Acoust. Soc. Am..

[9] Sakamoto S., Takakura R., Suzuki R., Katayama I., Saito R., Suzuki K. (2021). Theoretical and Experimental Analyses of Acoustic Characteristics of Fine-grain Powder Considering Longitudinal Vibration and Boundary Layer Viscosity. J. Acoust. Soc. Am..

[10] Sakamoto S., Ii K., Katayama I., Suzuki K. (2021). Measurement and Theoretical Analysis of Sound Absorption of Simple Cubic and Hexagonal Lattice Granules. Noise Control Eng. J..

[11] Allard J.F., Castagnede B., Henry M. (1994). Evaluation of tortuosity in acoustic porous materials saturated by air. Rev. Sci. Instrum..

[12] Tijdeman H. (1975). On the propagation of sound waves in cylindrical tubes. J. Sound Vib..

[13] Stinson M.R. (1991). The propagation of plane sound waves in narrow and wide circular tubes, and generalization to uniform tubes of arbitrary cross-sectional shape. J. Acoust. Soc. Am..

[14] Stinson M.R., Champou Y. (1992). Propagation of sound and the assignment of shape factors in model porous materials having simple pore geometries. J. Acoust. Soc. Am..

[15] Beltman W.M., van der Hoogt P.J.M., Spiering R.M.E.J., Tijdeman H. (1998). Implementation and experimental validation of a new viscothermal acoustic finite element for acousto-elastic problems. J. Sound Vib..

[16] Allard J.F. (1994). Propagation of sound in Porous Media Modeling Sound Absorbing Materials. J. Acoust. Soc. Am..

[17] Tabana E., Khavanina A., Jafarib A.J., Faridanc M., Tabrizid A.K. (2019). Experimental and mathematical survey of sound absorption performance of date palm fibers. Heliyon.

[18] Duan C., Cui G., Xu X., Liu P. (2012). Sound absorption characteristics of a high-temperature sintering porous ceramic material. Appl. Acoust..

[19] Sakamoto S., Higuchi K., Saito K., Koseki S. (2014). Theoretical analysis for sound-absorbing materials using layered narrow clearances between two planes. J. Adv. Mech. Des. Syst. Manuf..

